# Human seminal virome: a panel based on recent literature

**DOI:** 10.1186/s12610-022-00165-9

**Published:** 2022-09-06

**Authors:** Beatriz Helena Dantas Rodrigues de Albuquerque, Maryana Thalyta Ferreira Camara de Oliveira, Janaína Ferreira Aderaldo, Mychelle de Medeiros Garcia Torres, Daniel Carlos Ferreira Lanza

**Affiliations:** 1 Laboratório de Biologia Molecular Aplicada – LAPLIC, Departamento de Bioquímica, Centro de Biociências, Universidade Federal do Rio Grande do Norte, Natal, RN CEP: 59072-970 Brazil; 2 Rede Nordeste de Biotecnologia - RENORBIO, Universidade Federal do Rio Grande do Norte, Natal, RN Brazil; 3 Maternidade Escola Januário Cicco - Universidade Federal do Rio Grande do Norte, Natal, RN Brazil

**Keywords:** HPV, SARS-COV-2, HIV, HCV, HBV, infertility, semen, viral semen infection, HPV, SRAS-COV-2, VIH, VHC, VHB, infertilité, sperme, infection virale du sperme

## Abstract

**Background:**

The seminal virome and its implications for fertility remain poorly understood. To date, there are no defined panels for the detection of viruses of clinical interest in seminal samples.

**Results:**

In this study, we characterized the human seminal virome based on more than 1,000 studies published over the last five years.

**Conclusions:**

The number of studies investigating viruses that occur in human semen has increased, and to date, these studies have been mostly prospective or related to specific clinical findings. Through the joint analysis of all these studies, we have listed the viruses related to the worsening of seminal parameters and propose a new panel with the main viruses already described that possibly affect male fertility and health. This panel can assist in evaluating semen quality and serve as a tool for investigation in cases of infertility.

## Résumé

**Contexte:**

Le virome séminal et ses implications pour la fertilité restent mal compris. À ce jour, il n’existe pas de panels définis pour la détection des virus d’intérêt clinique dans les échantillons de sperme.

**Résultats:**

Dans cette étude, nous avons caractérisé le virome séminal humain sur la base de plus de 1000 études publiées au cours des cinq dernières années.

**Conclusions:**

Le nombre d’études portant sur les virus présents dans le sperme humain a augmenté et, à ce jour, ces études ont principalement été prospectives ou liées à des résultats cliniques spécifiques. Grâce à l’analyse conjointe de toutes ces études, nous avons répertorié les virus liés à l’aggravation des paramètres spermatiques, et nous proposons un nouveau panel avec les principaux virus déjà décrits qui peuvent affecter la fertilité et la santé masculines. Ce panel peut aider à évaluer la qualité du sperme et servir d’outil d’investigation dans les cas d’infertilité.

## Introduction

Infertility is a disease of the male or female reproductive system defined by the inability to establish a clinical pregnancy after 12 months or more of regular unprotected sexual intercourse [1]. It is estimated that infertility affects 8–12% of couples of reproductive age worldwide, with the male factor being solely responsible for 20–30% of the cases, contributing to 50% of records in general [2].

Infections and inflammatory reactions in the male genital tract (MGT) are among the main causes of infertility, accounting for 6–10% of the cases [3]. These infections are mainly caused by sexually transmitted pathogens and can induce infertility through multiple pathophysiological mechanisms, including impairment of seminal parameters and sperm functions [4, 5].

Various microorganisms, including bacteria, viruses, and protozoa, can infect the male reproductive tract and impair fertility [6]. Viral infections usually correspond to complex conditions, as there are no therapeutic measures for their control, and they can be transmitted causing infertility or subfertility in men [5]. Many viral families have strong tropism for the male reproductive tract, especially for the testes [7]. Salam and Horby [8] reported that 27 viral species can be found in human semen. In addition, severe acute respiratory syndrome coronavirus 2 (SARS-CoV-2) has been detected in human semen [9–11], raising concerns about the potential impact of this new coronavirus on male fertility.

In semen, viruses can infect sperm or sperm precursor cells, attach to molecules on the outside of sperm, present themselves as free virus particles, or reside in the seminal immune cells. Furthermore, viral infection of germ cells can result not only in changes in testicular function but also in the transmission of virus-induced mutations to subsequent generations [12].

Despite recent advances, there are no well-defined correlations regarding the effects of viral infections on fertility. This review aimed to identify and present the main recent findings about viruses in human semen, characterize the diversity of the seminal virome, identify the main viral species related to fertility, and discuss panels for viral identification that could have clinical applications and fertility research implications.

## Methodology

To characterize the human seminal virome, all studies published until May 8, 2021 available in the PubMed database were initially identified without language restriction. The search was performed using the following parameters: (“virology”[MeSH Subheading] OR “virology”[All Fields] OR “viruses”[All Fields] OR “viruses”[MeSH Terms] OR “virus s” [All Fields] OR “viruse”[All Fields] OR “virus”[All Fields]) AND (“semen”[MeSH Terms] OR “semen”[All Fields] OR “semen s”[All Fields] OR “semens “[All Fields] OR (“sperm s”[All Fields] OR “spermatozoa”[MeSH Terms] OR “spermatozoa”[All Fields] OR “sperm”[All Fields] OR “sperms”[All Fields]) OR “ seminal”[All Fields]).

To determine the main viruses that occur in semen, titles, abstracts, and full texts of articles published in the last 5 years were examined for discussion of the presence of the virus in semen by isolation or amplification and for the detection of nucleic acids or of specific antigens. Reviews, meta-analyses, and other publications that did not report the original clinical data were excluded. Studies conducted *in vitro* or in animal models and those with unavailable full texts were also not considered. Once the main viruses were identified, the search was expanded to include previously published articles and animal studies to deepen the discussion for each case.

## Results

**Fig. 1:**
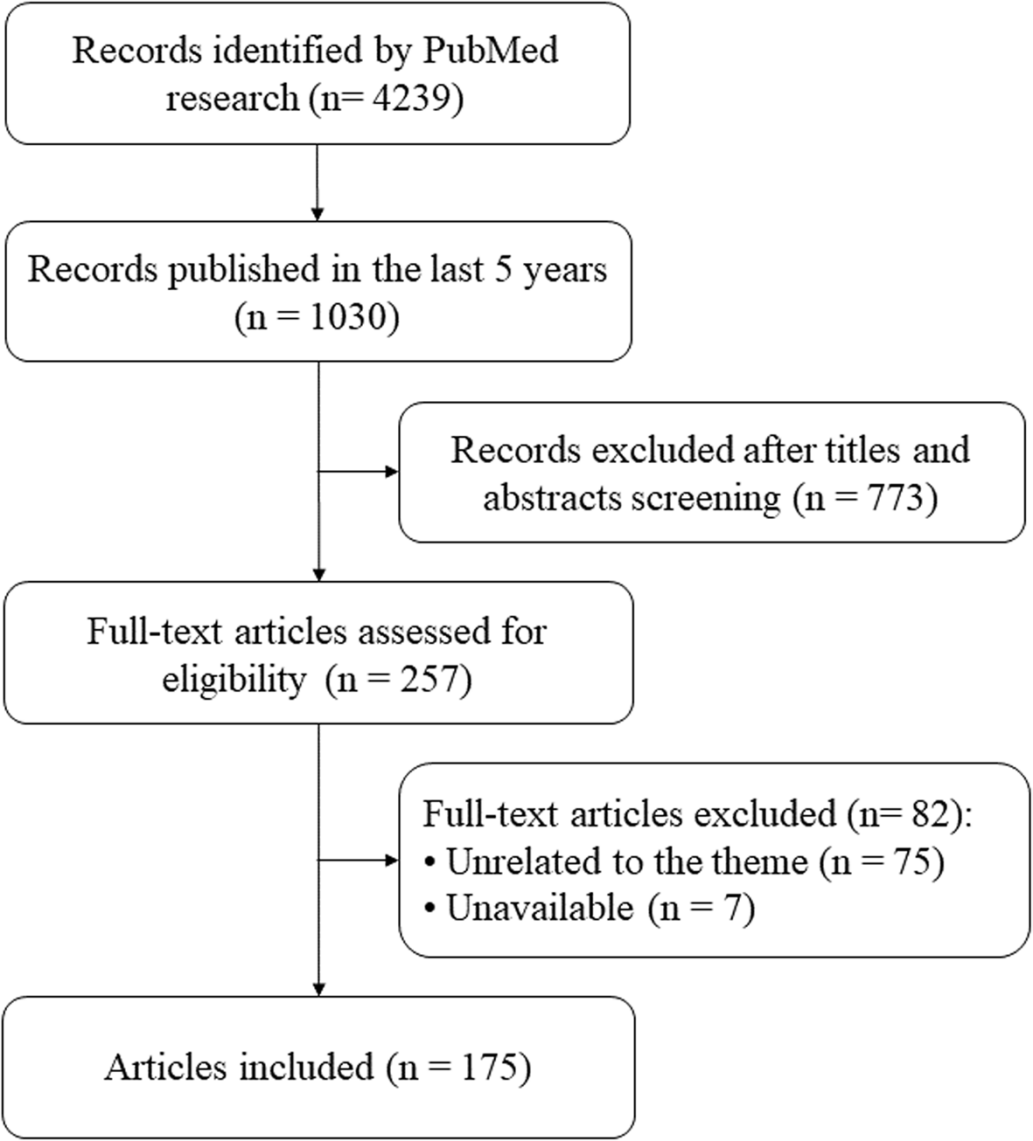
Flowchart of the literature review and study selection process

**Fig. 2:**
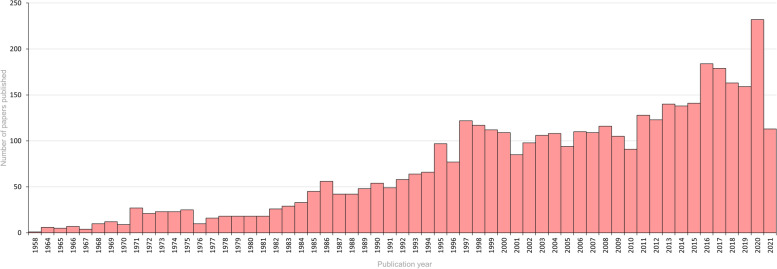
Number of articles related to viruses and semen published from 1958 to 2021. The X-axis corresponds to the year of publication and the Y-axis represents the number of articles published per year

We identified 4,239 articles published until May 08, 2021 (Fig. 1). Based on the number of articles published per year, it was observed that the number of studies concerning viruses and semen increased over time, becoming more evident from 2016 (Fig. 2). Given the considerable increase in the number of studies published in the last 5 years, we chose to consider only the articles published from 2016 onwards in this review, resulting in a total of 1,030 articles. After screening by title and abstract, 257 articles were selected, 75 were discarded because they were not directly related to the topic, and seven were not available in full. Thus, 175 articles (17%) met the eligibility criteria established for full text analysis.

**Fig. 3:**
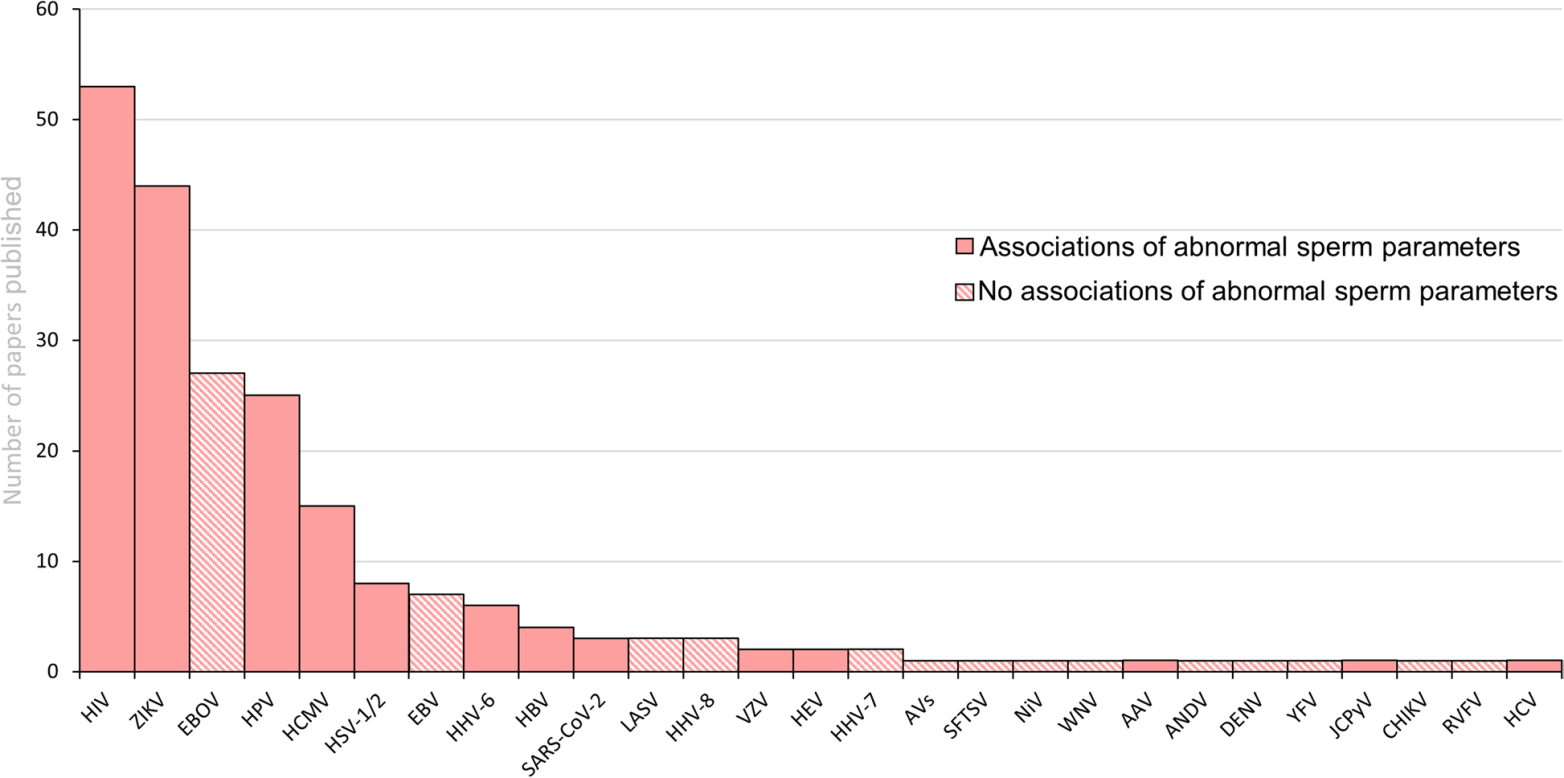
Overview of viruses detected in semen in this review and their associations with seminal parameters. The X-axis corresponds to the virus detected in human semen in the last five years, and the Y-axis represents the number of papers published. Abbreviations: HIV: Human immunodeficiency virus; ZIKV: Zika virus; EBOV: Ebola virus; HPV: Human papillomavirus; HCMV: Human cytomegalovirus; HSV 1/2: Herpes simplex virus type 1 and 2; EBV: Epstein-Barr virus; HVV-6: Human herpesvirus-6; HBV: Hepatitis B virus; SARS-CoV-2: Severe acute respiratory syndrome coronavirus 2; LASV: Lassa virus; HHV-8: Human herpesvirus-8; VZV: Varicella zoster virus; HEV: Hepatitis E virus; HHV-7: Human herpesvirus-7; AVs: Anelloviruses; SFTSV: Severe fever with thrombocytopenia syndrome virus; NiV: Nipah virus; WNV: West Nile virus; AAV: Adeno-associated virus; ANDV: Andes virus; DENV: Dengue virus; YFV: Yellow Fever Virus; JCPyV: JC polyomavirus; CHIKV: Chikungunya virus; RVFV: Rift Valley fever virus; HCV: Hepatitis C virus.

The analysis of selected articles revealed that 27 virus species were identified in human semen in the last 5 years, with the human immunodeficiency virus (HIV) being the most cited, followed by the Zika virus (ZIKV), Ebola virus (EBOV), human papillomavirus (HPV), and human cytomegalovirus (HCMV) (Fig. 3). Among the 27 viruses identified, 13 were associated with abnormalities in seminal parameters (Fig. 3). The main characteristics of each of the 27 viruses identified as well as a summary of the main effects already described on male reproductive health are presented in Table 1. The main information regarding the 13 viruses related to abnormalities in seminal parameters is described in the Discussion section.

**Table 1:** Characteristics, taxonomy, clinical presentation, and effects on male reproductive health of the viruses in semen

Virus	Family	Genus	Genome	Clinical presentation	Sexual transmission reported (Y/N/NA)	Effect on male reproductive health	References
HIV	*Retroviridae*	*Lentivirus*	ssRNA (RT)	Acquired Immunodeficiency Syndrome (AIDS)	Y	Orchitis, “Sertoli Cell only” syndrome, hypogonadism, abnormal sperm parameters and infertility	Teixeira et al., 2021 [7]; Dulioust et al., 2002 [13]; Nicopoullos et al., 2004 [14]; Bujan et al., 2007 [15]; Wong, Levy and Stephenson, 2017 [16]; Pudney and Anderson, 1991 [17]; Poretsky, Can and Zumoff, 1995 [18]; Shevchuk et al., 1999 [19].
ZIKV	*Flaviviridae*	*Flavivirus*	ssRNA (+)	Zika fever and congenital Zika leading to microcephaly and other central nervous system disorders	Y	Abnormal sperm parameters, orchitis, epididymo-orchitis, and infertility in mouse models	Teixeira et al., 2021 [7]; Govero et al., 2016 [20]; Vanegas et al., 2021 [21]; Huits et al., 2017 [22]; Joguet et al., 2017 [23]; Ma et al., 2016 [24]; Le Tortorec et al., 2020 [25].
EBOV	*Filoviridae*	*Ebolavirus*	ssRNA (-)	Ebola virus disease (EVD)	Y	Testis as an anatomic reservoir for persistence, erectile dysfunction and decreased libido	Teixeira et al., 2021 [7]; Guetiya et al., 2017 [26]; de St Maurice et al., 2018 [27]; Thorson et al., 2021 [28].
HPV	*Papillomaviridae*	*α-, β-, γ- Papillomavirus*	dsDNA	Warts and preneoplastic lesions associated to genital, anal and oropharyngeal cancers	Y	Anti-sperm antibody, abnormal sperm parameters, subfertility and infertility	Liu et al., 2018 [6]; Teixeira et al., 2021 [7]; Bezold et al., 2007 [29]; Garolla et al., 2013 [30]; Foresta et al., 2010 [31]; Connelly et al., 2001 [32]; Moghimi et al., 2019 [33]; Piroozmand et al., 2020 [34]; Moreno-Sepulveda and Rajmil, 2021 [35].
HCMV	*Herpesviridae*	*Cytomegalovirus*	dsDNA	CMV congenital infection and opportunistic infections	Y	Chronic inflammatory urogenital diseases, direct gametotoxic effect, can contribute to male infertility and have the potential to be transferred to the embryo after fertilization. Abnormal sperm parameters have also been reported	Gimenes et al., 2014 [5];Jahromi et al., 2020 [36]; Naumenko et al., 2011 [37]; Naumenko et al., 2014 [38]; Le Tortorec et al., 2020 [25].
HSV-1/2	*Herpesviridae*	*Simplexvirus*	dsDNA	Herpes labialis and genital herpes	Y	Prostatitis, epididymitis,urethritis, abnormal sperm parameters	Gimenes et al., 2014 [5]; Teixeira et al., 2021 [7]; Klimova et al., 2010 [39]; Wu et al., 2007 [40]; Kurscheidt et al., 2018 [41]; Monavari et al., 2013 [42]; Kapranos et al., 2003 [43]; Bradshaw et al., 2006 [44].
EBV	*Herpesviridae*	*Lymphocryptovirus*	dsDNA	Infectious mononucleosis, Burkitt lymphoma, nasopharyngeal carcinoma, and posttransplant lymphoproliferative disease	Y	Leukocytospermia	Neofytou et al., 2009 [45]; Le Tortorec et al., 2020 [25]; Bezold et al., 2001 [46].
HHV-6	*Herpesviridae*	*Roseolovirus*	dsDNA	Congenital infection, common childhood disease, exanthema subitum and acute febrile diseases	NA	Chronic inflammatory urogenital diseases and abnormal sperm parameters have also been reported	Naumenko et al., 2014 [38]; Salam and Horby, 2017 [8]; Le Tortorec et al., 2020 [25].
HBV	*Hepadnaviridae*	*Orthohepadnavirus*	dsDNA (RT)	Hepatitis, cirrhosis, and hepatocellular carcinoma	Y	Abnormal sperm parameters and infertility	Garolla et al ., 2013 [30]; Vicari et al ., 2006 [47]; Lee et al ., 2010 [48]; Lorusso et al ., 2010 [49]; Zhou et al ., 2011 [50]; Oger et al., 2011 [51].
SARS-CoV-2	*Coronaviridae*	*Betacoronavirus*	ssRNA (+)	Severe acute respiratory syndromeSevere acute respiratory syndrome	NA	Orchitis, hypogonadism and abnormal sperm parameters	Teixeira et al., 2021 [7]; Guo et al., 2021 [52]; Sengupta, Leisegang and Agarwal, 2021 [53].
LASV	*Arenaviridae*	*Mammarenavirus*	ssRNA (-) ambisense, segmentedssRNA (-) ambisense, segmented	Lassa fever	NA	Single case report of epididymitis	Salam and Horby, 2017 [8]; Le Tortorec et al., 2020 [25]; McElroy et al., 2017 [54]; Prescott et al., 2017 [55].
HHV-8	*Herpesviridae*	*Rhadinovirus*	dsDNA	Kaposi sarcoma, primary effusion lymphoma, multicentric Castleman’s disease and inflammatory cytokine syndrome	Y	Prostate cancer	Salam and Horby, 2017 [8]; Henning et al., 2017 [56]; Henning et al., 2017 [57]; Bellocchi, Svicher and Ceccherini-Silberstein, 2020 [58]; Bagasra et al., 2005 [59].
VZV	*Herpesviridae*	*Varicellovirus*	dsDNA	Chickenpox (varicella) and herpes zoster	NA	Abnormal sperm parameters was reported	Salam and Horby, 2017 [8]; Neofytou et al., 2009 [45]; Tavakolian et al., 2021 [60]; Ouwendijk et al., 2020 [61].
HEV	*Hepeviridae*	*Orthohepevirus A*	ssRNA (+)	Hepatitis and extrahepatic manifestations	NA	Abnormal sperm parameters was reported	Horvatits et al., 2021 [62]; Huang et al., 2018 [63]; Kamar et al., 2017 [64].
HHV-7	*Herpesviridae*	*Roseolovirus*	dsDNA	Exanthema subitum and status epilepticus with fever	NA	Unknown	Kaspersen et al., 2012 [65]; Bezold et al., 2001 [46]; Ljungman et al., 2008 [66].
AVs	*Anelloviridae*	14 genera have been identified	ssDNA	Unknown	NA	Unknown	Varsani et al., 2021 [67]; Li et al., 2020 [68]; Kaczorowska and Van Der Hoek, 2020 [69]; Martínez et al., 2000 [70].
SFTSV	*Phenuiviridae*	*Banyangvirus*	ssRNA (-)	Severe fever with thrombocytopenia syndrome	NA	Unknown	Kwak et al., 2019 [71]; Lee et al., 2019 [72]; Koga et al. 2019 [73].
NiV	*Paramyxoviridae*	*Henipavirus*	ssRNA (-)	Acute respiratory illness and fatal encephalitis	NA	Unknown	Aditi and Shariff, 2019 [74]; Arunkumar et al., 2018 [75].
WNV	*Flaviviridae*	*Flavivirus*	ssRNA (+)	Encephalitis and meningoencephalitis	NA	Single case report of orchitis	Le Tortorec et al., 2020 [25]; Gorchakov et al., 2019 [76]; Suthar, Diamond and Gale, 2013 [77]; Smith et al., 2004 [78].
AAV	*Parvoviridae*	*Dependoparvovirus*	ssDNA	Not known to cause disease. Used in virus-vectored gene-therapy trials	NA	Abnormal sperm parameters was reported	Behboudi et al., 2019 [79]; Rohde et al., 1999 [80]; Erles et al., 2001 [81]; Schlehofer et al., 2012 [82]; Le Tortorec et al., 2020 [25].
ANDV	*Hantaviridae*	*Orthohantavirus*	ssRNA (-)	Hantavirus cardiopulmonary syndrome, hantavirus pulmonary syndrome	NA	Unknown	Le Tortorec et al., 2020 [25]; Kuenzli et al. et al., 2018 [83].
DENV	*Flaviviridae*	*Flavivirus*	ssRNA (+)	Dengue fever, dengue haemorrhagic fever and dengue shock syndrome	NA	Unknown	Lalle et al., 2018 [84]; Whitehead et al., 2007 [85].
YFV	*Flaviviridae*	*Flavivirus*	ssRNA (+)	Yellow fever	NA	Autopsy revealed orchitis in some individuals	Duarte-Neto et al., 2019 [86]; Barbosa et al., 2018 [87]; Couto-Lima et al., 2017 [88].
JCPyV	*Polyomaviridae*	*Betapolyomavirus*	dsDNA	Almost exclusively in immunosuppressed individuals: progressive multifocal leukoencephalopathy	NA	Abnormal sperm parameters was reported	Rotondo et al., 2017 [89]; Comar et al., 2012 [90]; Le Tortorec et al., 2020 [25].
CHIKV	*Togaviridae*	*Alphavirus*	ssRNA (+)	CHIKV disease, arthralgia, myalgia	NA	Unknown	Le Tortorec et al., 2020 [25]; Bandeira et al. 2016 [91].
RVFV	*Bunyaviridae*	*Phlebovirus*	ssRNA (-), ambisense, segmented	Febrile disease, hemorrhagic fever, maculo-retinitis, encephalitis, miscarriage, hepatic and renal failure	NA	Unknown	Salam and Horby, 2017 [8]; Gregor et al., 2021 [92]; Haneche et al., 2016 [93].
HCV	*Flaviviridae*	*Hepacivirus*	ssRNA (+)	Hepatitis, cirrhosis, and hepatocellular carcinoma	Y	Abnormal sperm parameters, infertility and DNA damages.	Teixeira et al., 2021 [7]; Lorusso et al., 2010 [49]; Karamolahi et al., 2019 [94]; La Vignera et al., 2012 [95]; Hofny et al., 2011 [96]; Safarinejad, Kolahi and Iravani, 2010 [97]; Durazzo et al., 2006 [98].

*Abbreviations*: *HIV* Human immunodeficiency virus, *ZIKV* Zika virus, *EBOV* Ebola virus, *HPV* Human papillomavirus, *HCMV* Human cytomegalovirus, *HSV 1/2* Herpes simplex virus type 1 and 2, *EBV* Epstein-Barr virus, *HVV-6* Human herpesvirus-6, *HBV* Hepatitis B virus, *SARS-CoV-2* Severe acute respiratory syndrome coronavirus 2, *LASV* Lassa virus, *HHV-8* Human herpesvirus-8, *VZV* Varicella zoster virus, *HEV* Hepatitis E virus, *HHV-7* Human herpesvirus-7, *AVs* Anelloviruses, *SFTSV* Severe fever with thrombocytopenia syndrome virus, *NiV* Nipah virus, *WNV* West Nile virus, *AAV* Adeno-associated virus, *ANDV* Andes virus, *DENV* Dengue virus, *YFV* Yellow Fever Virus, *JCPyV* JC polyomavirus, *CHIKV* Chikungunya virus, *RVFV* Rift Valley fever virus, *HCV* Hepatitis C virus, *ssRNA (RT)* single-stranded RNA reverse-transcribing viruses, *ssRNA (+)* positive-sense single-stranded RNA viruses, *ssRNA (-)* negative-sense single-stranded RNA viruses, *dsDNA* double - stranded DNA viruses, *dsDNA (RT)* double-stranded DNA reverse-transcribing viruses. *Y*: Yes, *N* No, NA Not available

## Discussion

### HIV

Semen is the vector for most sexual transmissions of HIV worldwide. HIV can contaminate semen during the stages of acute and chronic infection and acquired immunodeficiency syndrome (AIDS) [99, 100]. The human immunodeficiency virus type 1 (HIV-1) may present in semen as free virions, associated with sperm, or virions that have invaded leukocytes [101]. The expression of the β-chemokine receptor C-C chemokine receptor type 5 (CCR5) in the peri-acrosomal region of the sperm surface and the presence of C-C chemokine receptor type 3 (CCR3) in the post-acrosomal cap could be involved in HIV-1 adhesion to spermatozoa, enabling these cells to act as virion carriers during sexual transmission of HIV-1 [5]. Leukocytes, including lymphocytes, monocytes, and macrophages, are considered the main vectors of HIV-1 in the semen [6].

The HIV viral load in seminal fluid is generally lower than that in blood [7]. However, HIV shedding persists in the semen of a subset of individuals who receive antiretroviral therapy, indicating that MGT may constitute a viral reservoir [100, 102]. This persistence is associated with different factors, including sexually transmitted infections (STIs), viral load in the blood, co-infection with HCMV or Epstein-Barr virus (EBV), and seminal cytokine levels [103–107].

The effects of HIV infection on seminal parameters can be observed in both asymptomatic and symptomatic patients [108]. However, the sperm abnormalities found in HIV patients are poorly understood, as both viral and antiretroviral treatments can cause changes [108, 109]. Semen alterations in HIV-infected men include decreased motility, sperm concentration, total sperm count, and ejaculate volume. In addition to this, abnormal morphology and a high percentage of sperm with DNA damage indicates impaired spermatogenesis [13, 110, 111].. The hypothesis is that decreased motility is related to mitochondrial toxicity caused by the nucleotide reverse transcriptase inhibitors used in therapy [112].

Furthermore, testis morphology and spermatogenesis are affected by disease progression [113]. Likewise, AIDS patients may develop chronic orchitis and, consequently, progressive hypergonadotropic hypogonadism, suggesting impaired testicular steroidogenesis [7].

### ZIKV

ZIKV can enter the testicular microenvironment, disrupt cellular metabolism, alter testicular physiology, and activate an intense immune response that can result in severe testicular damage and infertility [114].

Several studies have demonstrated that ZIKV is detected in the semen of infected men [115–118] up to six months after infection [119]. Although the reported persistence of ZIKV varies from days to months after the onset of symptoms, it is widely accepted that viral RNA persists longer and has a higher viral load in semen than in other bodily fluids. These observations suggest that ZIKV has tropism for the MGT, which may act as a viral reservoir, possibly due to the immunological privilege of the testes [6, 120]. Consequently, pregnant women should protect themselves against mosquito bites and also ensure safe sexual intercourse with their partner during pregnancy. Sexual transmission of ZIKV is possible and the most important condition associated with the infection is microcephaly, which forms in fetuses [121]. *Ex vivo* human tissue studies have revealed that several cell types, including germ cells, Sertoli cells, Leydig cells, and testicular resident macrophages, are permissive to ZIKV infection [122–124]. Likewise, it has been shown that, in mice, ZIKV preferentially infects spermatogonia, primary spermatocytes, and Sertoli cells in the testis, resulting in cell death and destruction of the seminiferous tubules [20]. In addition, there is evidence that ZIKV infection is associated with acute and chronic prostatitis in mouse and non-human primate models [125].

Sperm concentration and motility percentage may be significantly lower in the semen of ZIKV-positive individuals [21]. In addition to oligospermia, increased leukocytospermia, hematospermia, painful ejaculation, and penile secretion in patients with ZIKV infection, suggestive of local inflammation and tissue damage, have also been reported [22, 126]. Regarding strict Kruger morphology, a large percentage of sperm with head defects or various anomalies was also observed in ZIKV-positive samples [21, 23].

### HPV

HPV can be found anywhere in the male genital system, such as the external genitalia, urethra, prostate, epididymis, vas deferens, testes, and semen [127–132]. HPV DNA can be detected in sperm, somatic cells, and seminal plasma [133].

Several studies have reported that semen infection by HPV can interfere with different sperm parameters, such as count, vitality, motility and morphology, pH, semen viscosity, and the number of leukocytes can increase DNA fragmentation and the level of anti-sperm antibodies in semen [29–34].

The prevalence of seminal HPV infection is significantly higher in infertile men than in the general population [35]. It has been reported that HPV16, HPV51, HPV52, and HPV45 are the most frequently found genotypes [127, 129, 134]. HPV 16 appears to be the most frequent type, with a prevalence of 5.9% in the infertile population and 4.7% in the general population [35]. However, the prevalence of HPV genotypes can vary depending on the geographic area or country and additional factors, such as lifestyle or number of partners [135]. In addition, different semen fractions can contain multiple HPV types in varying amounts, with different HPV genotypes in the same fraction [136].

Infected sperms also serve as vectors for HPV transfer [133]. The penetration of HPV-infected sperm cells into oocytes results in the intracellular delivery of the HPV genome, followed by active transcription of the HPV genes in the fertilized egg [137, 138]. Perino et al. [134] reported that when HPV was present in semen, assisted reproduction techniques resulted in a lower fertilization rate and an increased percentage of abortions. Tangal et al. [139] also observed that after *in vitro* fertilization (IVF) treatment, implantation and pregnancy rates were similar in infected and uninfected males, but lower numbers of good-quality embryos and increased abortion rates were found in the presence of HPV-positive sperm.

The European Society of Human Reproduction and Embryology (ESHRE) Guideline on Viral Infection/Disease [140] also points to other studies that demonstrate the impact of seminal HPV infection on the results of assisted reproduction techniques. Among these, we highlight the following:

(1) Depuydt et al. [141] when investigating the clinical pregnancy rate of 732 couples, observed that the clinical pregnancy rate was significantly lower in women inseminated with HPV-positive semen (2.9% per cycle) than in those inseminated with HPV-negative semen (11.1% per cycle). Furthermore, above a ratio of 0.66 HPV virions/sperm, no pregnancies were observed.

(2) Garolla et al. [142] also reported that the cumulative pregnancy rate by intrauterine insemination and intracytoplasmic sperm injection (ICSI) for HPV-positive men was 14.2% (5/54) compared to that of 38.4% (66/172) for HPV-negative men, whereas the miscarriage rate was significantly higher in HPV-positive than in HPV-negative men (62.5% vs. 16.7%).

(3) Depuydt et al. [143] also analyzed 514 sperm samples from donors from three different sperm banks for 18 different HPV types. Overall, 3.9% (20/514) of tested donor sperms were positive for HPV, with different prevalences among the three different sperm banks (3.6% bank A, 3.1% bank B, and 16.7% bank C). It was observed that when sperm from the HPV-positive donor was used, no clinical pregnancy resulted, whereas when HPV-negative donor sperm was used, the clinical pregnancy rate was 14.6%.

Emerging evidence indicates that HPV infection in men affects sperm parameters and can reduce pregnancy and increase abortion rates [140].

### HCMV

HCMV has already been isolated from several secretions, including semen, indicating that this virus can infect the MGT and that semen can act as a vector for viral propagation [5, 144].

Some studies have not found any association [39, 45, 79, 145] between the impact of HCMV on male fertility and seminal parameters, whereas others have observed a positive correlation [12, 36, 40].

Bezold et al. [29] observed that HCMV was the most frequently detected pathogen in the semen of patients with infertility. However, they did not observe a significant association between the presence of HCMV DNA and the sperm parameters. In contrast, Jahromi et al. [36], in a study carried out at the infertility center of the Ghadir Maternal Child Hospital, identified an estimated prevalence of HCMV in semen (18.6%). They observed a higher prevalence of HCMV in the semen of men with abnormal semen analysis and a significant reduction in sperm morphology and count in the presence of HCMV, which supports the hypothesis that HCMV has a negative impact on male fertility.

HCMV can attach to the sperm surface and infect immature germ cells, which then develop into mature HCMV-carrying sperm [5, 37]. Furthermore, Naumenko et al. [37] observed a considerable decrease in the number of immature germ cells, indicating that HCMV produces a direct gametotoxic effect that may contribute to male infertility.

### Herpes simplex virus (HSV)

Herpes simplex virus type 1 (HSV-1) and herpes type 2 (HSV-2) have been widely detected in human semen with varying frequencies [29, 41, 42, 45, 60]. Bai et al. [146] observed that 2–50% of infertile men were positive for HSV-1/2.

The sources of HSV seminal DNA remain to be clarified, but it is known that HSV-2 can be internalized into healthy, motile sperm and is likely to cause direct damage to male germ cells [147].

HSV infections are associated with abnormal sperm parameters and male infertility, indirectly resulting in immune responses [42, 146, 148]. A strong association has been reported between HSV infection and problems with seminal parameters, including hematospermia, oligospermia, and increased apoptotic cells [39–43, 149, 150].

A reduction in seminal volume and abnormal viscosity has also been reported in men infected with HSV-2, which indicates prostate dysfunction [41]. Bezold et al. [29] reported significantly reduced sperm concentration and motility in addition to lower citrate and neutral α-glucosidase concentrations in HSV-infected men, suggesting compromised epididymal and prostate function.

### Human herpesvirus-6 (HHV-6)

HHV-6 is frequently found in semen samples [29, 38, 45, 151], but its effect on male fertility is unclear. Specific binding of human herpesvirus 6 B (HHV-6B) to the sperm acrosome has been observed, suggesting the existence of a specific receptor for the virus [65]. Furthermore, a higher prevalence of HHV-6 in men with chronic inflammatory disease of the urogenital tract has been observed [38]. This suggests that HHV-6 may contribute to the etiology of these diseases, but it does not lead to infertility, as a correlation between the detection of HHV-6 and reduced sperm parameters has not been observed.

HHV-6 is the only human herpes virus that integrates into the germline [152, 153]. HHV-6 genomes are usually found on chromosomes close to the telomeric ends, probably facilitated by homologous recombination through repeat sequences that flank the viral genome [154, 155]. Thus, HHV-6 integrated into germ cells can be transmitted vertically from parents to children, leading to congenital HHV-6 infection [156]. Godet et al. [151] detected two semen samples with high HHV-6 viral loads, consistent with the presence of integrated HHV-6 chromosomes. In these samples, sperm parameters revealed abnormal sperm morphology and immobile sperms.

### Hepatitis B virus (HBV)

Several studies have reported a reduction in sperm parameters attributed to HBV infection of semen [30, 47–50]. Lorusso et al. [49] observed that sperm concentration, motility, morphology, and viability were significantly impaired in HBV-seropositive patients. Karamolahi et al. [94] also reported similar results, whereby men infected with HBV and hepatitis C virus (HCV) showed a decrease in total sperm count, liquefaction time, and sperm motility, in addition to having an impaired morphology.

In addition to affecting seminal parameters, HBV infection can also cause serious damage to sperm DNA [157], as the HBV genome can integrate not only into host hepatocytes but also into human sperm chromosomes and induce chromosomal aberrations [158]. In this context, Huang et al. [159], using fluorescence *in situ* hybridization (FISH), showed that the HBV genome integrated into sperm chromosomes can be vertically transmissible through germ cells, producing heritable effects. Furthermore, HBV infection can have mutagenic effects on sperm chromosomes, leading to genomic instability and chromosomal aberrations.

Moreover, HBV induces the generation of reactive oxygen species (ROS) and reduces the antioxidant capacity of sperm cells, leading to an increase in oxidative stress [5, 160–163]. Thus, an increase in ROS concentration in spermatozoa can result in loss of membrane integrity, mitochondrial damage, genome damage, and apoptosis [5, 164–166]. In addition, HBV-induced oxidative stress can also affect male fertility, as observed by Qian et al. [163], who reported that oxidative stress can lead to a reduction in seminal parameters. They observed that the concentration of ROS in the semen of infertile men was negatively correlated with seminal volume, pH, sperm density, motility, morphology, activation rate, and sperm vitality.

Male HBV infection may result in a lower success rate in assisted reproduction procedures. Men with chronic HBV infection have been observed to have a significantly higher risk of low fertilization rate after IVF. This leads to a slight decrease in the total number of embryos fertilized [51]. Zhou et al. [50] also concluded that HBV infection in men is associated with impaired intracytoplasmic sperm injection and embryo transfer outcomes as well as reduced sperm quality.

HBV can exert a considerable impact on male fertility, as noted by Su et al. [167], who reported an increased incidence and risk of infertility among men with HBV infection compared to men without HBV infection.

### Severe acute respiratory syndrome coronavirus 2 (SARS-CoV-2)

Li et al. [9] identified SARS-CoV-2 in the semen of six hospitalized patients with coronavirus disease 2019 (COVID-19), including four patients who were in the acute phase of infection and two patients who were in clinical recovery. Additionally, Machado et al. [168] reported the presence of SARS-CoV-2 viral RNA in the semen of 15 asymptomatic and mildly symptomatic COVID-19 patients.

Gacci et al. [169] tested urine samples collected before and after ejaculation and semen from 43 sexually active men who recovered from SARS-CoV-2 infection. In the present study, sperm parameters and seminal interleukin-8 (IL-8) levels were evaluated. Only three patients tested positive in at least one sample. After recovery from COVID-19, 25% of the men studied were oligo-crypto-azoospermic, and among the 11 men with semen deficiency, eight were azoospermic, and three were oligospermic. Additionally, 33 patients had pathological levels of IL-8 in their semen. Likewise, Holtmann et al. [170] reported that the concentration, progressive motility, and total sperm count of patients with moderate infection were significantly lower than those of the controls.

In contrast, Ma et al. [24] reported the absence of SARS-COV-2 in semen samples from 12 patients with COVID-19. However, 33% of the population studied had altered seminal parameters and a greater number of sperms with DNA fragmentation. Thus, SARS-CoV-2 can activate cellular oxidative stress, leading to sperm DNA fragmentation, which is correlated with impaired embryonic development, lower implantation rates, and higher abortion rate [52].

Yang et al. [171] investigated whether SARS-COV-2 is present in 12 postmortem testis samples from COVID-19 patients. They reported the absence of SARS-COV-2 in the testis in 90% of cases. However, the testes of all patients exhibited significant seminiferous tubular injury, reduced number of Leydig cells, swelling, vacuolization, cytoplasmic rarefaction, detachment of the tubular basement membranes of Sertoli cells, and mild lymphocytic inflammation, corresponding to symptoms of orchitis. Likewise, Achua et al. [172] analyzed testicular specimens at the autopsies of six men with COVID-19 and relevant comorbidities. Three of the COVID-19 cases had impaired spermatogenesis, and only one case reported macrophage and lymphocyte infiltration into testicular tissues. Thus, orchitis and impaired spermatogenesis are possible complications of COVID-19 infection. Furthermore, other studies have reported that most male participants with COVID-19 have reduced testosterone levels, suggesting hypogonadism [173, 174].

Overall, these studies suggest that the occurrence of SARS-CoV-2 in semen is a rare event because of the small number of positive samples analyzed and the absence of viral RNA in semen [175–179]. However, previous studies have indicated that COVID-19 can have a negative impact on spermatogenesis and male fertility [53].

### Varicella-zoster virus (VZV)

The number of reports on the influence of VZV on semen is small, and these studies are controversial. Behboudi et al. [79] reported that 2.8% of the semen samples were positive for VZV DNA, but no significant difference was found between the seminal parameters of positive and negative samples. Neofytou et al. [45] detected VZV DNA in four semen samples, one with normozoospermia and three with sperm alterations. They also reported that VZV could be identified in both the sperm fraction and seminal fluid. In addition, a statistically significant difference was observed between VZV infection and teratospermia. Likewise, Tavakolian et al. [60] detected VZV in two semen samples from men with teratozoospermia.

### Hepatitis E virus (HEV)

The presence of HEV RNA in semen suggests that HEV can infect MGT and cause testicular damage [180]. Horvatits et al. [62] reported the presence of hepatitis E genotype 3 (HEV-3) viral particles in the ejaculate of immunocompromised men with chronic infection. In that study, HEV-3 was detected at much higher concentrations in the semen than in the blood, demonstrating HEV-3 replication in the male reproductive system. Furthermore, viral RNA concentrations were comparable in both fractions of the ejaculate, which may indicate that HEV-3 originates in the testes and prostate. In view of this, the authors concluded that MGT may be a niche for HEV-3 persistence in chronic infections.

HEV infection has also been described in the semen of infertile men. Huang et al. [63] reported a high prevalence of HEV RNA in the semen of infertile Chinese males. In this study, among patients with oligospermia, 53.57% were positive for HEV RNA, and more than 60% of sperm from patients infected with HEV were immotile. Additionally, changes in sperm morphology and vitality were observed. Thus, owing to oligospermia, asthenospermia, and necrozoospermia in HEV-infected men, the authors concluded that HEV infection impairs seminal quality.

Phylogenetic analyses indicated that all HEV isolates belonged to genotype 4 (HEV-4), the dominant strain in China. In contrast, El-Mokhtar et al. [181] reported discrepant results, in which HEV RNA was detected in the urine of patients with acute hepatitis E, but not in the serum. They also did not observe any changes in seminal quality. Horvatits et al. [182] reported no association between male infertility and HEV-3 infection.

### Adeno-associated virus (AAV)

AAV DNA is detected significantly more frequently in semen samples from infertile men than in normal semen samples [30]. Rohde et al. [80] reported, for the first time, the presence of AAV DNA in semen in 30% of samples from infertile men and absence in fertile men, suggesting that the presence of AAV in semen can affect sperm motility. Likewise, Erles et al. [81] detected AAV DNA in 38% of ejaculates from men with alterations in seminal parameters (oligoasthenozoospermia or asthenzoospermia) and in 4.6% of semen samples without alterations. The same study also detected AAV DNA in 10 of 38 testicular tissue biopsies from infertile men and in two of eight orchidectomy samples.

In a study by Schlehofer et al. [82], in infertile couples, the presence of AAV DNA was observed in 14.9% of cervical smears and 19.9% of semen samples. However, no significant association with fertility was observed. In addition, there was no evidence of sexual transmission of AAV. Furthermore, Behboudi et al. [79] detected AAV DNA in 27.6% of semen samples, but no association was found between seminal AAV infection and semen quality.

### JC polyomavirus (JCPyV)

The shedding of JCPyV in urine has been commonly reported and this virus has also been detected in prostate tissue [183–186]. Few studies report the presence of JCPyV in human semen [89, 90, 187].

Rotondo et al. [89] identified JCPyV DNA in semen samples with an overall prevalence of 27.6%. Likewise, Comar et al. [90] also reported a higher prevalence of JCPyV sequences in semen (24.5%) and urine (43.4%) samples from infertile men than in those form the control group. This study was the first to indicate an association between JCPyV and male infertility. A reduction in sperm motility was observed in 84.6% of the positive samples for JCPyV, whereas 76.9% had altered sperm morphology. However, further investigations are needed to better understand the possible role of JCPyV in male infertility, as well as its persistence in semen and its capacity for sexual transmission.

### HCV

Several studies have demonstrated a negative impact of HCV on seminal quality [49, 95–98]. In a clinical evaluation of 82 HCV patients aged between 18 and 60 years, the mean total sperm count and the levels of normal motility and morphology were significantly lower than those of control subjects. Likewise, a significantly higher frequency of disomy for chromosomes 18, X, and Y was observed in men with chronic hepatitis C than in the control subjects. Baseline serum levels of luteinizing hormone (LH), follicle-stimulating hormone (FSH), and testosterone were also significantly lower [97].

Karamolahi et al. [94] observed that men infected with HBV and HCV had reduced sperm counts, progressive motility, and normal sperm morphology. Furthermore, Moretti et al. [188] reported not only a lower fertility index but also higher sperm diploidy in individuals with seminal HCV infections, suggesting that apoptosis and sperm necrosis play important roles in these patients. Similarly, an analysis of 40 patients with chronic hepatitis C infection and primary infertility and another 20 patients with HCV infection and secondary infertility demonstrated that progressive motility and sperm morphology were significantly impaired in these patients compared to those in the controls. It was also observed that sperm mitochondrial membrane potential, chromatin compaction, and sperm DNA fragmentation were significantly altered. In addition, seminal levels of ROS and viral replication correlated with a worsening of seminal parameters [95].

### Ebola virus (EBOV)

The testis is likely to be an anatomical reservoir for EBOV persistence in humans [7]. Although the exact mechanism of viral tropism has not yet been determined, it has been hypothesized that the persistence of EBOV is established in the interstitium of the male reproductive tract (seminal vesicle, epididymis, prostate gland, and testis) and is shuttled to the seminal fluid via infected tissue macrophages [189, 190].

To date, the longest time from acute Ebola virus disease (EVD) illness to the detection of viral RNA in a semen sample is 40 months [191]. Furthermore, sexual transmission from male survivors to female partners has been identified up to 470 days after the illness offset [192]. Therefore, the World Health Organization updated its guidelines for the prevention of EBOV transmission in 2016 to include practicing safe sex and hygiene for 12 months from the onset of symptoms or until two negative semen tests for EBOV were reported [193].

However, because of logistical challenges in affected countries and biosafety considerations related to laboratory manipulation of EVD, much of the pathophysiology of viral persistence in semen has been overlooked [194]. Sexual health complaints such as erectile dysfunction and decreased libido are commonly reported among EVD survivors [26, 27, 120]. Nevertheless, the causal mechanisms underlying these complaints remain unclear. Although physiologic conditions can play a role, it is also likely that psychosocial factors including residual stress, trauma, stigma, and grief contribute as well [120].

### Panel for detection of viruses associated with male infertility

**Fig. 4:**
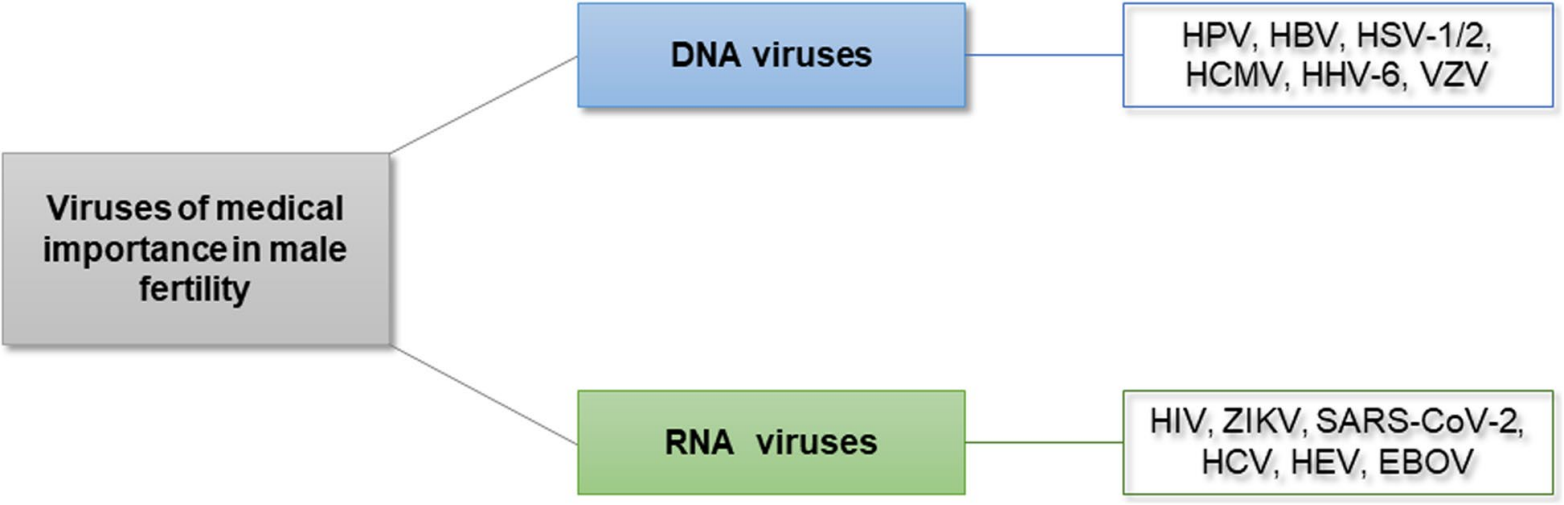
Panel of viruses of medical importance to be analyzed in infertile men. Abbreviations: HPV, human papillomavirus; HBV, hepatitis B virus; HSV1/2, herpes simplex virus type 1 and 2; HCMV, human cytomegalovirus; HVV-6, human herpesvirus-6; VZV, varicella-zoster virus; HIV, human immunodeficiency virus; ZIKV, zika virus; SARS-CoV-2, severe acute respiratory syndrome coronavirus 2; HCV, hepatitis C virus; HEV, hepatitis E virus; EBOV, Ebola virus

Based on the data available to date, we propose a panel that includes the main viruses affecting the quality of human semen (Fig. 4).

HPV was the first virus included in this panel, as it is usually present in semen samples [34, 129, 195, 196]. Furthermore, according to Boeri et al. [197], accurate investigation of the presence of seminal HPV in the diagnostic analysis of infertile men is of paramount importance, not only for its potential negative pathophysiological impact on male fertility but also in terms of the general health of men.

HBV can integrate its DNA into the genome of male germ cells [5], which raises safety issues regarding paternal-fetal transmission in men with chronic hepatitis B, especially in assisted reproduction procedures, such as ICSI. Therefore, the Belgian–Dutch Association for Artificial Insemination advises against ICSI treatment for chronic patients with HBV [50]. Condijts et al. [198] suggested that strategies to select sperm cells without HBV incorporation are necessary to avoid excluding chronically infected men.

HCV can be transmitted during IVF [199]. Thus, sequential semen preparation with density gradient centrifugation followed by swim-up is recommended for HCV-positive men. Likewise, if one of the partners is chronically infected, therapy should be considered before fertility treatment to reduce viral load [5]. Thus, seminal detection of HCV is essential, both to verify the success of antiviral therapy in the seminal elimination of HCV in these patients and to minimize the risk of cross-transmission.

The Herpesviridae family, composed of eight members (HSV-1, HSV-2, VZV, EBV, HHV-6–8, and HCMV), is considered one of the main risk factors for infertility [60]. In this review, we observed that HSV-1, HSV-2, HCMV, and HHV-6 are herpesviruses that present a greater risk for male fertility and recommend the investigation of these four pathogens.

HIV-infected men, in addition to having alterations in seminal parameters, may also intermittently release HIV-1 RNA into the seminal plasma during antiretroviral therapy, even with undetectable RNA in blood plasma [102, 200, 201]. Thus, semen washing by density gradient centrifugation followed by sperm swim-up has been used as an option for serodiscordant couples who wish to become pregnant when the man is infected with HIV [5, 202, 203]. Therefore, molecular investigation of HIV in semen can be used to verify the success of seminal lavage and the safe use of clinical specimens, as well as to assess the efficiency of antiviral treatment.

Likewise, owing to the global spread and lack of knowledge about its potential effect on male fertility and embryonic and fetal development, investigation of the presence of SARS-CoV-2 in semen is necessary as a preventive measure to ensure greater protection of assisted reproductive technologies and assessment of male infertility.

ZIKV and EBOV are also of great importance for the assessment of fertility, as they can infect not only the testis but also several male genital organs that act as viral reservoirs [25]. However, these viruses can be included in this panel depending on the epidemiological scenario in each region. For example, frequent outbreaks of EBOV in the African continent have been reported, which, in this context, justify its investigation, due to the persistent elimination of EBOV in semen. Likewise, arboviruses have great epidemiological importance in Latin America, and as ZIKV is a virus that can reduce sperm quality and affect male fertility, in addition to having a potential impact on fetal development, its investigation is of great relevance.

## Conclusions

Based on the data collected here, it is possible to propose a new panel of viruses that affect seminal quality. This panel is composed of HPV, HBV, HSV-1/2, HCMV, HHV-6, VZV, HIV, ZIKV, SARS-Cov-2, HCV, HEV, and EBOV. This set of viruses could be a starting point for the development of different methods for quality semen screening and diagnosis, contributing to the standardization of viral identification kits. In this way, the implementation of this panel will improve the quality control of semen, allowing a more accurate diagnosis for counseling infertile couples.

## Data Availability

All data generated or analyzed during this study are included in this published article and its supplementary information files.

## Abbreviations

AAV: Adeno-associated virus
AIDS: Acquired immunodeficiency syndrome
ANDV: Andes virus
AVs: Anelloviruses
CCR3: C-C chemokine receptor type 3
CCR5: C-C chemokine receptor type 5
CHIKV: Chikungunya virus
COVID-19: Coronavirus disease 2019
DENV: Dengue virus
EBOV: Ebola virus
EBV: Epstein-Barr virus
ESHRE: European Society of Human Reproduction and Embryology
EVD: Ebola virus disease
FISH: Fluorescence *in situ* hybridization
FSH: Follicle-stimulating hormone
HBV: Hepatitis B virus
HCMV: Human cytomegalovirus
HCV: Hepatitis C virus
HEV: Hepatitis E virus
HEV-3: Hepatitis E virus genotype 3
HEV-4: Hepatitis E virus genotype 4
HHV-6: Human herpesvirus-6
HHV-6B: Human herpesviruses 6B
HHV-7: Human herpesvirus-7
HHV-8: Human herpesvirus-8
HIV: Human immunodeficiency virus
HIV-1: Human immunodeficiency virus type 1
HPV: Human papillomavirus
HPV16: Human papillomavirus 16
HPV45: Human papillomavirus 45
HPV51: Human papillomavirus 51
HPV52: Human papillomavirus 52
HSV: Herpes simplex virus
HSV-1: Herpes simplex virus type 1
HSV-2: Herpes simplex virus type 2
ICSI: Intracytoplasmic sperm injection
IL-8: Interleukin-8
IVF: *In vitro* fertilization
JCPyV: JC polyomavirus
LASV: Lassa virus
LH: Luteinizing hormone
MGT: Male genital tract
NiV: Nipah virus
ROS: Reactive oxygen species
RVFV: Rift Valley fever virus
SARS-CoV-2: Severe acute respiratory syndrome coronavirus 2
SFTSV: Severe fever with thrombocytopenia 96 syndrome virus
STIs: Sexually transmitted infections
VZV: Varicella zoster virus
WNV: West Nile virus
YFV: Yellow Fever virus
ZIKV: Zika virus
